# Prebiotics Modulate the Effects of Antibiotics on Gut Microbial Diversity and Functioning *in Vitro*

**DOI:** 10.3390/nu7064480

**Published:** 2015-06-04

**Authors:** Laura P. Johnson, Gemma E. Walton, Arianna Psichas, Gary S. Frost, Glenn R. Gibson, Timothy G. Barraclough

**Affiliations:** 1 Department of Life Sciences, Imperial College London, Silwood Park Campus, Ascot, Berkshire, SL5 7PY, UK; E-Mail: L.johnson12@imperial.ac.uk; 2 Food Microbial Sciences Unit, Department of Food and Nutritional Sciences, The University of Reading, Reading, Berkshire RG6 6AH, UK; E-Mails: g.e.walton@reading.ac.uk (G.E.W.); g.r.gibson@reading.ac.uk (G.R.G.); 3 Section of Investigative Medicine, Division of Diabetes, Endocrinology and Metabolism, Imperial College London, Hammersmith Hospital, London W12 0HS, UK; E-Mails: ap811@cam.ac.uk (A.P.); g.frost@imperial.ac.uk (G.S.F.)

**Keywords:** gut microbiota, antibiotics, prebiotics, fibre

## Abstract

Intestinal bacteria carry out many fundamental roles, such as the fermentation of non-digestible dietary carbohydrates to produce short chain fatty acids (SCFAs), which can affect host energy levels and gut hormone regulation. Understanding how to manage this ecosystem to improve human health is an important but challenging goal. Antibiotics are the front line of defence against pathogens, but in turn they have adverse effects on indigenous microbial diversity and function. Here, we have investigated whether dietary supplementation—another method used to modulate gut composition and function—could be used to ameliorate the side effects of antibiotics. We perturbed gut bacterial communities with gentamicin and ampicillin in anaerobic batch cultures *in vitro*. Cultures were supplemented with either pectin (a non-fermentable fibre), inulin (a commonly used prebiotic that promotes the growth of beneficial bacteria) or neither. Although antibiotics often negated the beneficial effects of dietary supplementation, in some treatment combinations, notably ampicillin and inulin, dietary supplementation ameliorated the effects of antibiotics. There is therefore potential for using supplements to lessen the adverse effects of antibiotics. Further knowledge of such mechanisms could lead to better therapeutic manipulation of the human gut microbiota.

## 1. Introduction

The human colon contains many hundreds of species of bacteria, which collectively play an important role in digestion and health. A growing body of work demonstrates the importance of the gut microbiota for digesting long-chain molecules in the diet [1], synthesising key nutrients [2], protecting against colonisation by pathogenic bacteria, priming the immune system [3], and stimulating hormonal pathways that affect various aspects of host physiology [4]. One key challenge for targeting the gut microbiota for improved health is how to manage this complex system, in which benefits derive from the actions of an assemblage of many species, rather than a single agent.

Several methods are available for manipulating the gut microbiota. Antibiotics are widely used to control pathogenic bacteria. However, antibiotics can have an adverse effect on gut communities by reducing the abundance and diversity of commensal microbes [5,6]. There is therefore a need to identify ways to reduce the adverse impacts of antibiotics on gut communities. Increased knowledge about ecological responses, and how those may affect key metabolic functions, would help design such approaches.

Another way to manipulate the microbiota is to modify resource supply, either via a controlled diet or the addition of dietary supplements. One such approach that promotes the growth of bacteria beneficial to human health is the use of prebiotics [7]. Diet plays a major role in shaping gut communities [8], and many beneficial functions of gut bacteria relate to the fermentation of non-digestible macromolecules.

Non-digestible carbohydrates (fibre), including starch, are the main nutrient source for colonic bacteria [9]. Bacteria ferment fibre to produce short chain fatty acids (SCFAs) [10], mainly acetate, propionate and butyrate [11]. SCFAs afford multiple benefits for the host, including providing an energy source for organs and triggering hormone pathways [12]. SCFAs activate free fatty acid receptor 2 (FFA2), located on the luminal surface of colonic L-cells [13], which induces secretion of anoretic gut hormones such as peptide YY (PYY). PYY is released into the circulation and induces anorexigenic signalling, which leads to decreased food intake [11]. Dietary intervention to stimulate SCFA production and thereby regulate anoretic gut hormones offers a potential strategy to combat the current obesity epidemic [14]. High fibre diets and prebiotics such as inulin are known to stimulate growth of beneficial bacteria such as *Bifidobacterium*, and to reduce appetite and the risk of obesity [15,16]. However, exactly how these effects are mediated by changes in the microbial community remains unclear.

Here, we explore whether dietary supplementation can be used to reduce the adverse effects of antibiotic treatment on gut diversity and functioning. If so, this might help to devise refined interventions combining antibiotic and prebiotic use. We measured the interactive effects of antibiotics and dietary supplements on faecal microbial composition and SCFA production in anaerobic batch cultures. We monitored bacterial composition by Illumina-sequencing the V4 region of the 16S rRNA gene. We also tracked counts of total bacteria, and *Bacteroides* and *Bifidobacterium*, two of the main taxa involved in fermenting carbohydrates, over 30 h using fluorescence *in situ* hybridisation (FISH). *Bacteroides* and *Bifidobacterium* were chosen to study as they comprise a significant component of the gut microbiota, and were expected to differ in their responses to both dietary supplements and antibiotics. SCFA concentrations were determined using gas chromatography (GC) [17]. In order to determine potential effects of observed changes in bacterial communities on host physiology, we also used an *in vitro* assay to measure PYY release.

Ampicillin and gentamicin were used to perturb the bacterial community. Ampicillin targets both Gram-negative and Gram-positive bacteria, whereas gentamicin targets mainly Gram-negative species. Most *Bacteroides* spp. (Gram-negative) are resistant to both ampicillin (through production of beta-lactamase) [18] and gentamicin (because they do not transport the molecule to the ribosomal target site) [19], although levels of resistance vary among isolates. In contrast, *Bifidobacterium* spp. (Gram-positive) are widely susceptible to ampicillin but resistant to gentamicin [20,21]. We supplemented cultures using two non-digestible carbohydrates, pectin and inulin. Pectin is a complex polysaccharide that forms part of the plant cell wall and is found in many plant-based foods [22]. It is well fermented by many bacteria in the gut, especially *Bacteroides* [9]. We chose pectin as a simple treatment to examine the effects of a high fibre *versus* a low fibre diet. Inulin, also a non-digestible carbohydrate, is a widely used prebiotic [23] that is also found naturally in many plants including artichoke and asparagus. It stimulates the growth of beneficial bacteria such as bifidobacteria [24].

## 2. Experimental Section

### 2.1. Faecal Sample Preparation

Faecal samples used in this study came from healthy human volunteers with no history of gastrointestinal illness, who were not regular consumers of pre or probiotics and had not consumed antibiotics within the previous 4 months. One volunteer was used per experiment (3 volunteers used overall). Faecal samples were donated on the morning of the experiment, and kept under anaerobic conditions for use within 30 min. 15–20 g faeces were diluted 1:10 with pre-reduced phosphate buffered saline (PBS). The solution was homogenised in a Stomacher 400 circulator (Seward) for 2 min. The resulting faecal slurry was used to start batch cultures.

### 2.2. Batch Culture Fermentation

Batch culture fermentation vessels contained 135 mL sterile basal medium (per litre: 2 g peptone water, 2 g yeast extract, 0.1 g NaCl, 0.04 g K_2_HPO_4_, 0.04 g KH_2_PO_4_, 0.01 g MgSO_4_·7H_2_O, 0.01 g CaCl_2_·6H_2_O, 2 g NaHCO_3_, 2 mL Tween 80, 0.05 g hemin, 10 μL vitamin K, 0.5 g l-Cysteine HCl, 0.5 g bile salts and 4 mL resazurin solution). A nitrogen gas supply was connected to the vessels to sustain an anaerobic environment, and vessels were left to gas overnight. pH was maintained at 6.8 via pH electrodes between pH 6.7–6.9 (the distal large intestine has a neutral pH), and temperature maintained at 37 °C via a circulating water bath. The following day, each vessel was inoculated with 15 mL faecal slurry (1% final concentration). Ampicillin and gentamicin (25 mg) were added to appropriate vessels, at concentrations representative of the amount that would typically make it to the large intestine. The effect of fermentable fibre was investigated via the addition of pectin (1.5 g), which emulated a high fibre diet (The control medium represented a low fibre diet). Effects of prebiotic supplementation were examined via the addition of inulin (1.5 g). Samples were collected from each vessel over a series of time points (0, 5, 10, 24, 30 and 48 h) and prepared for further assays. Bacterial enumeration was carried out using flurorescence *in situ* hybridization (FISH), as described by Costabile *et al.* [25]. Probes were commercially synthesised and fluorescently labelled using cyanine 3 (Cy3). Targeted groups of bacteria were total bacteria, *Bacteroides*, and *Bifidobacterium*, using probes EUBmix [26], Bac303 [27] and Bif164 [28] respectively. 4,6-diamidino-2-phenylindole (DAPI) nucleic acid stain was used to visualise all bacteria under an ultra violet (UV) light. The experiment was repeated three times, with a different donor each time.

### 2.3. DNA Extraction and 16S Sequencing

Samples of 1.5 mL collected from batch culture fermentation vessels were centrifuged at 13,000 g for 10 min, supernatant was removed and the remaining pellet resuspended in 0.5 mL PBS glycerol (for freezing at −20 °C). After thawing, samples were centrifuged at 13,000 *g* for 5 min. The supernatant was discarded and the pellet resuspended in 1 mL PBS. Samples were centrifuged once more, and pellets were resuspended in 750 μL bead solution and transferred to bead beating tubes (as supplied). DNA extraction was then carried out using Power Fecal DNA isolation kit (#12830-50, MO BIO Laboratories) according to manufacturer’s instructions. The 16S rRNA V4 variable region was sequenced using the 515/806 primer set with barcode on the forward primer. PCR ran with 30 cycles using the HotStarTaq Plus Master Mix Kit (Qiagen, Valencia, CA, USA): 94 °C for 3 min; 28 cycles of 94 °C for 30 s, 53 °C for 40 s and 72 °C for 1 min; and an elongation step at 72 °C for 5 min. Illumina TruSeq DNA libraries were prepared using pooled PCR product purified using calibrated Ampure XP beads. PCR and sequencing was performed at MR DNA (www.mrdnalab.com, Shallowater, TX, USA) on a MiSeq machine following manufacturer’s guidelines. Sequences were processed using a proprietary analysis pipeline (MR DNA, Shallowater, TX, USA): Barcodes and chimeras (sequences shorter than 150 bp and those with ambiguous base calls) were removed. Operational taxonomic units (OTUs) were defined by clustering at 3% divergence (97% similarity) and taxonomically classified using BLASTn against the GreenGenes database [29]. Focusing on the top fifteen most frequent genera across all samples, we used the frequency (% of sequences belonging to a particular taxon) of each genus (and of all other genera combined) as our data for taxonomic composition. We used principal components analysis (PCA) on these data to derive major access of variation in genus frequencies, using the prcomp function in R [30].

### 2.4. SCFA Analyses

Samples of 1.5 mL collected from batch culture fermentation vessels were centrifuged at 13,000 g for 10 min. Supernatants were collected, filtered using a 0.22 μm syringe filter (PVDF) and stored at 4 °C. A standard stock solution was prepared for each acid (acetic, propionic, iso-butyric, butyric, iso-valeric, valeric and caproic acid) at a concentration of 100 mM. A 10 mM master mix was prepared (1 mL of each 100 mM standard, 3 mL H_2_O) with a final volume of 10 mL. This was serially diluted to create standard concentrations of 1 mM, 100 μM, 10 μM, 1 μM, 100 nM and 10 nM. An internal standard solution was also prepared; 132.8 μL ethyl-butyrate was added to 9.87 mL H_2_O to create a 100 mM ethyl-butyrate solution. The internal standard was created at a final volume of 10 mL: 100 μL 100 mM ethyl-butyrate was added to 337 μL formic acid, followed by H_2_O. Internal standard and sample were then added to vials (225 μL of each). All standards and samples were analysed using a GC method. Analysis was performed using Nukol Capillary Column (30 m × 0.23 mm × 1.0 μm), (SUPELCO Analytical, UK). Injector and detector temperatures were both 250 °C, and helium was used as the carrier gas at a flow rate of 5.0 mL min^−1^. Concentrations of SCFAs present within samples were then calculated.

### 2.5. PYY Release Assays

We followed the methods of Frost *et al.* [31] described in detail in Supplementary Methods 1. In brief, colons were taken from 6 C57BL6 mice (in total). Isolated colonic cells were applied to 1% Basement Membrane Matrix (BD Matrigel, VWR)- coated 24-well plates. Following washing, cells were then incubated with faecal fluids obtained from anaerobic batch cultures for 2 h at 37 °C. Propionate was used as a positive control as previous work has shown that it significantly stimulates PYY release [32]. Secretion buffer alone was used to estimate basal release of PYY from colonic L-cells. Supernatants and lysates were then collected from each well and PYY concentration was measured by radioimmunoassay (RIA).

### 2.6. Statistical Analyses

We ran linear mixed effects models of log(concentration) for each SCFA in turn, including diet (as short-hand for dietary supplementation), antibiotic, time and their interactions as fixed effects and microcosm as a random effect (to take account of pseudo-replication due to repeated measures). Inspection of the plots revealed a saturating response in most cases, so we included time^2^ into the model. Inspection of outputs of models showed that time^2^ was not significant for interaction terms, so we refitted a model with diet * antibiotic * time + time^2^ as our fixed effects. Analysis of variance (ANOVA) confirmed that this formulation did not lead to significant reduction in explanatory power for all SCFAs. To examine whether there were significant differences in the effects of inulin *versus* pectin, we refitted the model with an alternative factor coded as “control diet” *versus* “supplemented diet”, *i.e*., making no distinction between supplements. If this alternative model was not significantly worse than the model using separate levels for each diet, we simplified and refer to effects of “diet supplementation” without referring separately to inulin or pectin. A similar procedure was used to test whether the two antibiotics had different effects.

## 3. Results

### 3.1. Starting Composition

**Figure 1:**
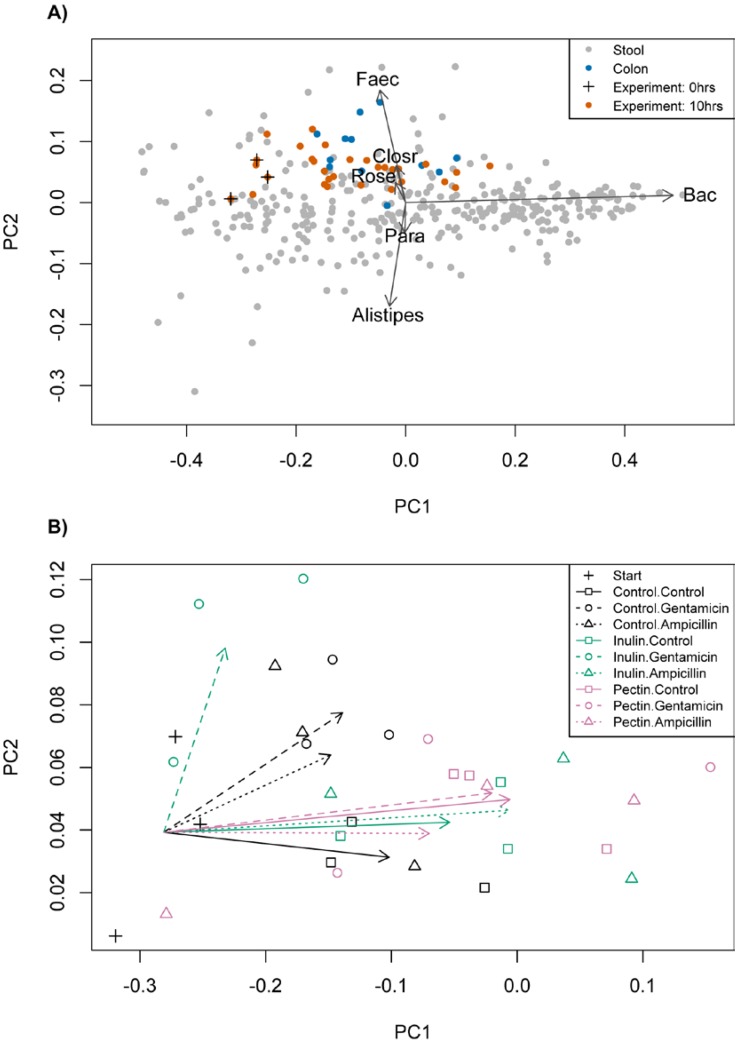
(**A**) The first two principal components representing variation in frequencies of common genera across Human Microbiome Project samples (grey), stool samples from Stearns *et al.* [35] (also grey), colon samples from Stearns *et al.* [35] (blue), our starting cultures (marked by +) and our time 10 experimental samples (orange). Correlations with six main genera are shown. (**B**) An expanded plot focusing on our experimental samples, with arrows showing the change in mean values of principal component 1 (PC1) and principal component 2 (PC2) in each experimental treatment. *Faecalibacterium* (Faec), *Clostridium* (Closr), *Roseburia* (Rose), *Bacteroides* (Bac), *Parabacteroides* (Para).

The taxonomic composition of cultures at the start (T0) was based on >50,000 reads of the 16S V4 region per sample and fell within variation observed in the Human Microbiome Project (HMP) (Figure 1A). All communities had low/medium *Bacteroides* (17.9%–24.0%), high *Faecalibacterium* (12.7%–15.9%) and high *Ruminococcus* (5.4%–12.3%) frequencies relative to variation across HMP samples [33], which corresponds to enterotype 3 in Arumugam *et al.* [34]. Over 10 h, cultures became more similar to communities sampled from colon biopsies (blue dots, Figure 1A), indicated by a significant shift along the major axis of variation, corresponding mainly to increased frequency of *Bacteroides* (mean change in principal component 1 (PC1) = 0.195, *t* = 6.22, conservative d*f* = 2, *p* = 0.025; Figure 1B). This shows that the composition of our faecal communities is similar to the composition in the colon, and therefore provides support that our artificial system is representative of the gut microbiota.

### 3.2. Changes in Bacterial Abundance

**Figure 2:**
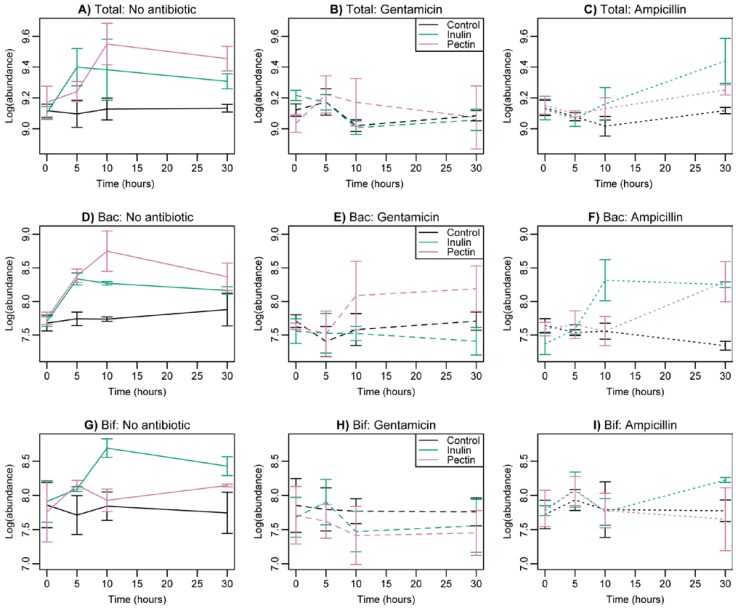
Changes in the abundance of total bacteria (**A**) to (**C**), *Bacteroides* (**D**) to (**F**) and *Bifidobacterium* (**G**) to (**I**) as determined by FISH counts during the course of the experiment. Columns show results with no antibiotics ((**A**, **D**, **G**), solid lines), gentamicin ((**B**, **E**, **H**), dashed lines) and ampicillin ((**C**, **F**, **I**), dotted lines) in turn. Standard error bars are shown. Black = control; green = inulin; red = pectin. Total (total bacterial abundance), *Bacteroides* (Bac), *Bifidobacterium* (Bif). Averages across volunteers are shown.

Supplementation with inulin and pectin led to a significant increase in total bacterial numbers relative to control medium (Figure 2A, F_2,22_ = 3.86, *p* = 0.037, Table 1). Antibiotic treatment decreased cell numbers. The impact of gentamicin was sustained over 30 h (Figure 2B), whereas ampicillin reduced numbers over 10 h, but growth recovered later in the experiment (Figure 2C, interaction between antibiotic and quadratic time, F_4,75_ = 4.58, *p* = 0.0023, Table 1). There was no significant interaction between the effects of fermentable fibre and antibiotics on total cell counts.

**Table 1:** Linear mixed effects model of bacterial cell counts over time. The best models included time as a quadratic term for total bacteria and *Bacteroides*, and time as a factor for *Bifidobacterium*.

Response Variable	Explanatory Term	Sum Squares	Mean Squares	Degrees of Freedom	*F*	*p*
Total bacteria	Diet	0.159	0.079	2.22	3.86	0.0365
	Antibiotic	0.239	0.120	2.22	5.81	0.0094
	Time	0.087	0.044	2.75	2.12	0.1274
	Antibiotic:Time	0.377	0.094	4.75	4.58	0.0023
*Bacteroides*	Diet	0.668	0.334	2.27	6.34	0.0055
	Antibiotic	1.006	0.503	2.27	9.54	0.0007
	Time	1.949	0.975	2.81	18.48	<0.0001
	Diet:Antibiotic	0.355	0.089	4.27	1.68	0.1826
	Diet:Time	1.399	0.350	4.81	6.63	0.0001
	Antibiotic:Time	0.730	0.183	4.81	3.46	0.0116
	Diet:Antibiotic:Time	1.954	0.244	8.81	4.63	0.0001
*Bifidobacterium*	Diet	0.048	0.024	2.27	0.70	0.5043
	Antibiotic	0.156	0.078	2.27	2.29	0.1202
	Time	0.291	0.097	3.81	2.85	0.0423
	Diet:Antibiotic	0.087	0.022	4.27	0.64	0.6381
	Diet:Time	0.438	0.073	6.81	2.15	0.0565
	Antibiotic:Time	0.957	0.160	6.81	4.70	0.0004
	Diet:Antibiotic:Time	1.100	0.092	12.81	2.70	0.0041

We also measured abundances of two key taxa, *Bacteroides* and *Bifidobacterium* spp. using taxon-specific FISH markers. *Bacteroides* displayed a significant interaction between fermentable fibre, antibiotics and time (F_8,81_ = 4.63, *p* = 0.0001, Table 1). Supplementation with pectin and inulin increased growth in the absence of antibiotics (Figure 2D). Supplementation with pectin reduced the adverse effects of gentamicin after 10 h, whereas inulin reduced the effects of ampicillin (Figure 2E,F). By 30 h, however, both supplements reduced the effects of ampicillin. Only gentamicin and inulin continued to have a negative effect on bacterial abundance after 30 h.

*Bifidobacterium* spp. also displayed a significant interaction between fermentable fibre, antibiotics and time (with time as a factor, F_12,81_ = 2.70, *p* = 0.004, Table 1). After 10 h, supplementation with inulin led to higher counts in the absence of antibiotics (Figure 2G), but this effect was removed by the addition of both antibiotics (Figure 2H,I). After 30 h, supplementation with both pectin and inulin increased counts in the absence of antibiotics, but the effect was negated by gentamicin (both pectin and inulin) and ampicillin combined with pectin. By 30 h, however, there was no longer a negative effect of ampicillin on cultures supplemented with inulin (Figure 2I).

### 3.3. Taxonomic Composition

We focused on the 10 h time point to measure taxonomic composition in more detail using Illumina 16S V4 sequencing, as bacteria should be in the exponential phase of growth. Variation in taxonomic composition was summarised using the same principal components identified in Section 3.1 and Figure 1A. The use of fermentable fibre in the absence of antibiotics led to an increase in scores of both PC1 and PC2, (Figure 1B, *F*_2,6_ = 5.46, 5.68; *p* = 0.045, 0.042 in turn). Increased PC1 primarily indicates a higher proportion of *Bacteroides* whereas increased PC2 indicates a higher proportion of *Faecalibacterium* and changes in several genera (Figure 1A, examined further below). Adding antibiotics to the basal medium alone led to communities with higher scores for PC2 (Figure 1B, *F*_2,6_ = 14.55, *p* = 0.0051). We looked separately at changes in the most frequent 15 genera plus the combined frequency of all remaining taxa, which we call ‘other genera’. The change in PC2 corresponded especially to increased frequency of *Faecalibacterium*, *Ruminococcus*, *Alistipes*, *Roseburia*, and ‘other genera’, and decreased *Parabacteroides* and the proteobacteria, *Shigella* and *Escherichia* (Figure S1, Table S1). The only marginal differences between the two antibiotics were that *Allisonella* increased with gentamicin but decreased with ampicillin, and *Roseburia* increased further with gentamicin than ampicillin. Factoring in variation in total counts (*i.e.*, an increase in frequency could still be a decrease in abundance if total counts decline), the only significant changes were declines in *Bacteroides*, *Parabacteroides*, *Shigella* and *Escherichia* in the presence of antibiotics (Table S2).

When applied in combination, there were significant interactions in the effects of fermentable fibre and antibiotic on taxonomic composition along PC2 (*F*_4,15.9_ =3.64, *p* = 0.027). The effect of ampicillin was reduced by supplementation with fermentable fibre, whereas that of gentamicin was reduced by pectin, but amplified by inulin (Figure 1B). Among component taxa, *Faecalibacterium*, *Alistipes* and *Roseburia* all displayed significant interactions between fermentable fibre and antibiotics, and their responses were highly correlated across treatments (Figures S1 and S2, Table S3). Several other genera also showed responses correlated with these taxa and matched the response in PC2 (Figue S2): Antibiotics led to increased frequencies in the absence of fibre, little change with pectin, and either little change (ampicillin) or an amplified increase (gentamicin) with inulin. *Bacteroides* displayed a negative relationship with the responses of these taxa: For example, we observed an increase with pectin and a decrease with inulin when gentamicin was added (Figures S1 and S2).

We checked the congruence between frequencies estimated by FISH and by 16S sequencing at 10 h. There was a significant positive correlation for *Bacteroides* (*t* = 3.79, d*f* = 23.7, *p* = 0.0009) and responses across treatments were congruent (Figure S3), although the interaction term between fermentable fibre and antibiotic was significant with FISH but not with Illumina (*F*_4,16_ = 3.25, *p* = 0.040 and *F*_4,18_ = 1.45, *p* = 0.25 respectively). There was no significant relationship between FISH and Illumina frequencies for *Bifidobacterium* across cultures overall (*t* = 0.64, d*f* = 23.2, *p* = 0.53), and estimates were far lower for Illumina than FISH. Responses across treatments were incongruent between the two measures (Figure S3). *Bifidobacterium* is known to be underrepresented with standard primers [36,37]. For this reason, we attach more weight to the FISH results than 16S results for this genus.

### 3.4. Short Chain Fatty Acids

SCFA production, as measured by GC, varied considerably among vessels and over time, ranging from 1.4 to 67.0 mM. Supplementation with fermentable fibre increased acetate production significantly over time (*F*_1,73.5_ = 8.86, *p* = 0.004, Figure S4). In the absence of fermentable fibre, gentamicin increased acetate production marginally while ampicillin decreased it. Fermentable fibre had no effect on acetate production in the presence of gentamicin, but increased acetate production with ampicillin (Figure S4, interaction between fermentable fibre and antibiotics, *F*_2,21.3_ = 4.05, *p* = 0.032). The main trends, therefore, were that gentamicin removed the effects of fermentable fibre, but supplements restored acetate to control levels in the presence of ampicillin. Propionate displayed similar patterns across treatments to acetate production. Butyrate levels also varied similarly, except that the effects of fermentable fibre with gentamicin became significantly more negative over time (Figure S4, *F*_2,68_ = 4.89, *p* = 0.010). This pattern was also found with isobutyrate, and became even more pronounced with valerate, isovalerate (both C5) and caproate (C6). Supplementation with pectin and inulin reduced production by 30 h (Figure S4).

**Figure 3:**
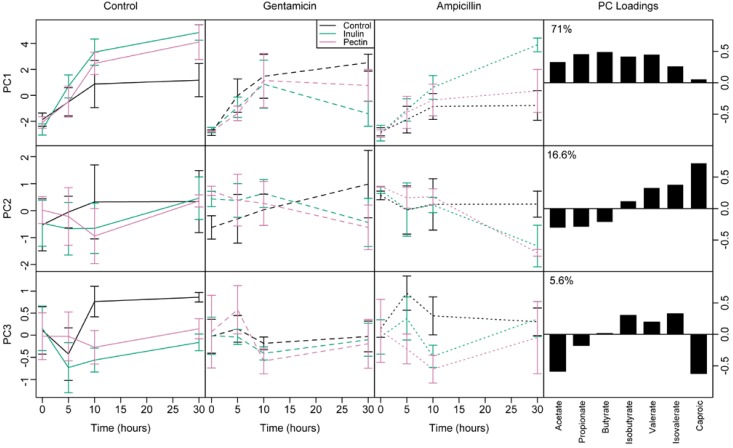
Changes through time of the first three principal components summarising variation in short chain fatty acids (SCFAs) log(concentrations). Black = control; green = inulin; pink = pectin. Solid = control antibiotics; dashed=gentamicin; dotted = ampicillin. The loadings and % variation for each principal component are shown in right-hand panels. Principal component 1 (PC1) represents production of all SCFAs except caproate. Principal component 2 (PC2) represents increasing concentration of SCFAs of longer chain length. Principal component 3 (PC3) represents proportionately increased concentration of BCFAs relative to acetate and caproate.

To identify independent component of responses, we ran a principal components analysis, then modeled the effects of fermentable fibre and antibiotic on the first 3 components, which together described 93.2% of the total variation (Figure 3). PC1 represented overall production of SCFAs, as it was positively correlated with all SCFAs (Figure 3). Supplementation with fermentable fibre increased PC1 (overall production of SCFAs) with no antibiotic treatment and ampicillin treatment, but decreased PC1 over time with gentamicin (antibiotic by diet by time interaction, *F*_2,75.7_ = 5.70, *p* = 0.005). Interestingly, PC2 represented a gradient from low to high carbon number: High values indicate higher concentrations of longer chain and lower concentrations of shorter chain SCFAs (Figure 3, mid right panel). Dietary supplements had little effect on PC2 (gradient from low to high carbon number) with no antibiotic treatment, whereas PC2 increased over time with gentamicin on the control diet, but decreased in both pectin and inulin treatments (antibiotic by diet by time interaction, *F*_2,75_ = 5.81, *p* = 0.0045). This indicates proportionately greater production of shorter chain fatty acids (acetate, propionate, butyrate) relative to longer chain fatty acids (valerate, isovalerate and caproate) caused by supplements combined with antibiotic treatments. Finally, principal component 3 (PC3) represented proportionately greater production of branched chain fatty acids (BCFAs) isobutyrate and isovalerate relative to acetate and caproate. Carbohydrate supplementation reduced PC3 (BCFA production) irrespective of antibiotic treatment (*F*_2,26.6_ = 5.92, *p* = 0.022), indicating proportionately fewer BCFAs in supplemented vessels.

### 3.5. Effect of Microbial Abundance and Composition on SCFA Production

We re-ran models to test whether the densities of total bacteria, *Bacteroides* or *Bifidobacterium* from FISH counts could explain variation in SCFA production (Table 1). We added each bacterial variable in turn into the best models for each SCFA and PC1 to PC3 identified above. Additional variation in PC1 was weakly predicted by *Bacteroides*, PC2 by total bacteria, and PC3 by none of the bacterial measures. The main pattern therefore is that relatively more SCFAs are produced when there are more bacterial cells.

Focusing on time 10, we looked for correlations between SCFA production and the frequencies of different genera, as represented by taxon PC1 and PC2. First, we refitted just the effects of diet and antibiotics at time 10 alone. Henceforth, we refer to the principal components from the analysis of taxon frequencies as TAXON.PC1 and TAXON.PC2, and the principal components from the analysis of SCFAs as SCFA.PC1, SCFA.PC2, and SCFA.PC3. SCFA.PC1 correlated solely with antibiotics, SCFA.PC2 did not vary significantly among treatments, and SCFA.PC3 varied solely with diet. We then tested whether taxon composition, represented by TAXON.PC1 and TAXON.PC2 in turn, explained additional variation in any of the SCFA.PCs. SCFA.PC2 was significantly greater (*i.e.*, producing , longer chain fatty acids) when TAXON.PC1 was greater (signifying a lower proportion of *Bacteroides*, *t* = −3.10, d*f* = 23.1, *p* = 0.005). We searched for additional correlations with specific genera for the other two components of SCFA variation. SCFA.PC1 was significantly lower (*i.e.*, lower overall SCFA production) with higher frequencies of *Escherichia* (*t* = −2.82, 21.3, *p* = 0.01). No taxa were correlates of SCFA.PC3. Note, that these tests involved multiple comparisons, finding a significant correlation with one taxon is not unlikely even under the null model of no true associations, but we present the correlations in a spirit of hypothesis generation rather than testing.

### 3.6. PYY

We were unable to assess the effects of antibiotics on PYY release. A lactate dehydrogenase (LDH) assay indicated that faecal fluid samples that contained antibiotics were cytotoxic to colonic L-cells (Figure S5A). Both antibiotics had significant cytotoxic effects, but neither pectin nor inulin supplementation led to any cytotoxicity (Figure S5B). Incubation of colonic L-cells with faecal fluid samples caused a significant increase in PYY release compared to the secretion buffer control (Figure S5B). However, there was no significant difference between cultures supplemented with either fibre source and unsupplemented cultures.

Linear mixed effect models revealed some significant correlations between taxa and PYY release. *Bifidobacterium* (Illumina frequencies) correlated positively with the release of PYY from colonic L-cells (*t* = 3.36, *p* = 0.006). *Bacteroides* (FISH counts) was also found to correlate positively with PYY release (*t* = 3.38, *p* = 0.008). However, Illumina frequencies for *Bacteroides* were not significant. Results also revealed some metabolic correlations: BCFAs isobutyrate (*t* = −3.8, *p* = 0.01) and valerate (*t* = −2.7, *p* = 0.04) had a significant negative relationship with PYY release.

## 4. Discussion

Antibiotics and fermentable fibres interacted in their effects on the composition and metabolic functioning of faecal communities. The effect of each fibre depended on which antibiotic was present and vice versa. In some cases, fermentable fibre restored growth and/or metabolic function reduced by antibiotic treatments, in other cases antibiotics negated the benefits of fermentable fibre. We discuss each response type in turn, before returning to general implications for manipulating gut microbiota for improved metabolic function.

Whole faecal communities were cultured in anaerobic batch cultures. Whilst batch conditions do not perfectly mimic the gut environment, they were designed to mimic resource availability and physical conditions in the colon. Indeed, our comparison with colon biopsies supported that our system is representative of the gut microbiota. Total bacterial growth was minimal in the absence of fermentable fibre, supplementation with both fermentable fibres resulted in increased bacterial growth as additional resources were present. As expected, *Bacteroides* spp. increased in abundance when pectin was added, and *Bifidobacterium* spp. increased when inulin was added. *Bacteroides* spp. also increased with inulin supplementation (though not as much as with pectin). Inulin fermentation is not ubiquitous within *Bacteroides*, but some species such as *B. thetaiotaomicron* can ferment it [38].

Total bacterial abundance declined with both antibiotics when fermentable fibre was absent. Gentamicin had lasting effects, whereas the abundance of cultures treated with ampicillin recovered after 30 h. This might reflect the growth of ampicillin-resistant taxa using resources made available by the loss of ampicillin-susceptible taxa; or there may have been *in situ* evolution of ampicillin-resistance, for example by mutation in the penicillin binding protein [39]. Alternatively, this may be due to degradation of the antibiotic by 30 h, however this still reflects realistic changes expected following a dose of antibiotics *in vivo*. Intriguingly, the effects of antibiotics on focal taxon abundances did not mirror those predicted from the literature. Contrary to the expectation of resistance to both antibiotics, *Bacteroides* declined with gentamicin in cultures supplemented with inulin, and with ampicillin in cultures supplemented with pectin (although growth with ampicillin had recovered by 30 h). These differences could reflect differential growth of sub-populations of *Bacteroides* able to break down inulin, such as *B. thetaiotaomicron* [38], although they are not expected to be competitive when bifidobacteria are present. Alternatively, the responses could reflect indirect effects from increases or decreases in other taxa that either facilitate or inhibit growth of each taxon [40]. For example, an *in vivo* study of the combined effects of ampicillin and gentamicin found reduced abundance of *Bacteroides*, even though most should be resistant to both drugs [41]. Ampicillin led to the expected decline in *Bifidobacterium* abundance with both pectin and inulin, although growth was restored with inulin after 30 h. Unexpectedly, however, gentamicin reduced growth with both pectin and inulin. Again, this could reflect indirect effects caused by changes in other taxa that facilitate or compete with *Bifidobacterium*. Alternatively, inulin might enhance growth of beta-lactamase secreting taxa, which could reduce concentrations of ampicillin over time with benefits for all taxa.

Similar effects were observed across multiple genera surveyed by 16S sequencing. In control medium, application of either antibiotic led to a shift towards lower values of PC1 and higher values of PC2. Notably, this was indicative of increased frequency of *Faecalibacterium*. An increase in *Faecalibacterium* frequency has been observed in *ex vivo* faecal communities treated with ampicillin previously [42], and as a Gram-positive Firmicute, it is expected to be insensitive to gentamicin. As seen with FISH data, the effects of antibiotics were modulated by fermentable fibre: Both supplements removed the effects of ampicillin on bacterial abundance, whereas only pectin removed the effects of gentamicin. Inulin actually amplified changes caused by gentamicin, associated with even greater reduction in *Bacteroides* and increase in *Faecalibacterium* frequency. The increase in *Faecalibacterium* can readily be explained by the well-known use of inulin by this genus [43,44], although here the effect was only detected when gentamicin was added. We cannot explain this interaction based on our results, but it could relate to reduction of Gram-negative bacteria that would otherwise compete with *Faecalibacterium* for inulin.

Dietary supplements also altered the effects of antibiotics on metabolite production. Fibre supplementation increased the production of all three main SCFAs and reduced proportions of BCFAs, isobutyrate and isovalerate, which result from fermentation of protein [45]. Pectin and inulin are amongst the most highly fermented substrates in the gut and are known to stimulate production of unbranched SCFAs [12,17]. The responses to antibiotics mirrored changes in *Bacteroides* abundance across treatments, which indeed was the only taxon found to positively correlate with overall SCFA production (*Escherichia* also displayed a negative correlation with total SCFAs). Supplementation with both pectin and inulin also led to decreased concentration of longer chain fatty acids over time when either antibiotic was present. This component of variation in SCFA correlated with taxon PC1, which also mainly reflects the frequency of *Bacteroides*. *Bacteroides* taxa ferment polysaccharides to both acetate and propionate [46,47], but are not generally thought of as producing butyrate or longer-chain fatty acids. Definitively attributing changes in SCFA production to particular taxa was not possible here, due to the large number of taxa with correlated responses and the smaller number of repeated measures over time. Our results provide hypotheses for future testing.

We intended to look at the effects of fermentable fibre and antibiotics on PYY release. However, an LDH assay indicated that antibiotic treatment was cytotoxic to colonocytes. Although not usually toxic, colon cells were directly incubated with antibiotics in our culture system, which appeared to cause necrosis. However, we must remember that this is an *in vitro* method; the system lacks hormones, mucins and other physiological components that exist in the human colon. The remaining sample size was too small to reveal strong correlations with dietary supplementation. We found no direct effect of dietary supplementation or SCFA concentrations on PYY levels. *Bifidobacterium* frequency was positively correlated with the release of PYY, which matches evidence that rats fed with probiotic strains of *Bifidobacterium* exhibit increased plasma levels of PYY *in vivo* [48]. We also found that BCFAs isobutyrate and valerate had a negative effect on PYY release. Isobutyrate and valerate are typically products of fat and protein fermentation [49]. We report the results for completeness but future work with larger sample sizes would be needed to verify them. The next stage may be to repeat the study using humans, and conduct a feeding study.

## 5. Conclusions

In conclusion, addition of fermentable carbohydrates greatly affected the response of cultured communities to antibiotic treatments. Generally, the positive effects of fermentable fibre on bacterial growth and SCFA production were removed when antibiotics were applied. However, certain combinations of antibiotic and fermentable fibre were able to restore the bacterial community. Notably, inulin reduced the negative effects of ampicillin after 30 h, thus restoring metabolic function and taxonomic composition using prebiotics. This treatment combination suggests a potential role for prebiotics in restoring function during and after antibiotic treatment, which may have broader clinical impacts.

## Supplementary Files

Supplementary File 1
