# Supplementary material for: Prebiotics Modulate the Effects of Antibiotics on Gut Microbial Diversity and Functioning in Vitro
Source: Nutrients. 2015 Jun 4;7(6):4480–97. doi: 10.3390/nu7064480 (PMC4488797; doi:10.3390/nu7064480)
Supplement: Supplementary File 1 [file nutrients-07-04480-s001.docx]

**Supplementary Information**

**1. Supporting Methods**

*1.1. Isolation of Colonic Cells*

Colons were obtained from C57BL6 mice (≥8 weeks of age) and collected into Leibovitz media
(l-15, PAA). Colons were digested in Dulbecco’s Modified Eagle’s Media (DMEM) supplemented with 10% fetal calf serum (FCS), 1% Penicillin-Streptomycin and 0.4 mg mL^−1^ Collagenase-XI
(Sigma UK) at 37 °C for 15 min to liberate intestinal crypts. After a series of vigorous shaking/centrifugation steps to create a series of digests (until colon was completely homogenised), all digests were centrifuged once more at 300 g for 5 min. Supernatants were discarded and all pellets were resuspended and pooled together to create a final cell suspension. The cell suspension was then filtered through a nylon mesh (pore size 250 μm), and plated onto 24-well plates pre-coated with 1% Basement Membrane Matrix (BD Matrigel, VWR). The plates were incubated overnight at 37 °C in an atmosphere of 95% O_2_ and 5% CO_2_.

*1.2. Hormone Secretion*

Colonic cells were washed three times with secretion buffer (4.5 mM KCl, 138 mM NaCl,
4.2 mM NaHCO_3_, 1.2 mM NaH_2_PO_4_·2H_2_O, 2.6 mM CaCl_2_, 1.2 mM MgCl_2_, 10 mM HEPES in 1 L
glass-distilled water). The pH was adjusted to 7.4 using NaOH and supplemented with 0.1%
fatty-acid-free bovine serum albumin (BSA, Sigma). Faecal fluids were diluted in secretion buffer (1:5), cells were then incubated at 37 °C for 2 h with diluted faecal fluids (300 μL well^−1^). Secretion buffer alone represents basal release of PYY from colonic cells. Supernatants were collected from wells and centrifuged for 3 min (100 g). Pellets were discarded and supernatants were stored at −20 °C. The remaining cells were treated with 250 µL lysis buffer per well (40 mL glass distilled water (GDW),
0.25 g Na deoxycholic acid, 0.5 mL IGEPAL, 1.5 mL 5M NaCl, 2.5 mL 1M Tris HCL pH 8, 1 tablet of complete EDTA-free protease inhibitor cocktail (Roche)) and plates were stored at −80 °C overnight. Lysates were collected the following day. Each well was then washed with 250 µL secretion buffer and the contents of each well were then transferred into corresponding eppendorf tubes containing lysed cells.

*1.3. Radioimmunoassay (RIA)*

Synthetic peptide YY (PYY) at a concentration of 0.5 pmoL mL^−1^ was added to generate a standard curve for supernatants and lysed samples. Antiserum (Y21) was produced in rabbits against synthetic porcine PYY coupled to BSA by glutaraldehyde. Y21 primary antibody cross reacts fully with PYY and does not cross-react with other gut peptides. Radioactive antigen (^125^I-PYY) was used in the assays.
All assay tubes were vortexed and incubated at 4 °C for 3 days (protected from light). Immunoprecipitation was carried out using 100 µL sheep anti-rabbit secondary antibody, samples were incubated for 1 h at room temperature. 100 µL 10% polyethylene glycol (PEG) and 500 µL 0.1%
Triton X were then added. Tubes were centrifuged at maximum speed for 30 min at 4 °C to separate free antigen from bound antigen. Free and bound radioactivity was measured using a gamma
scintillation counter.


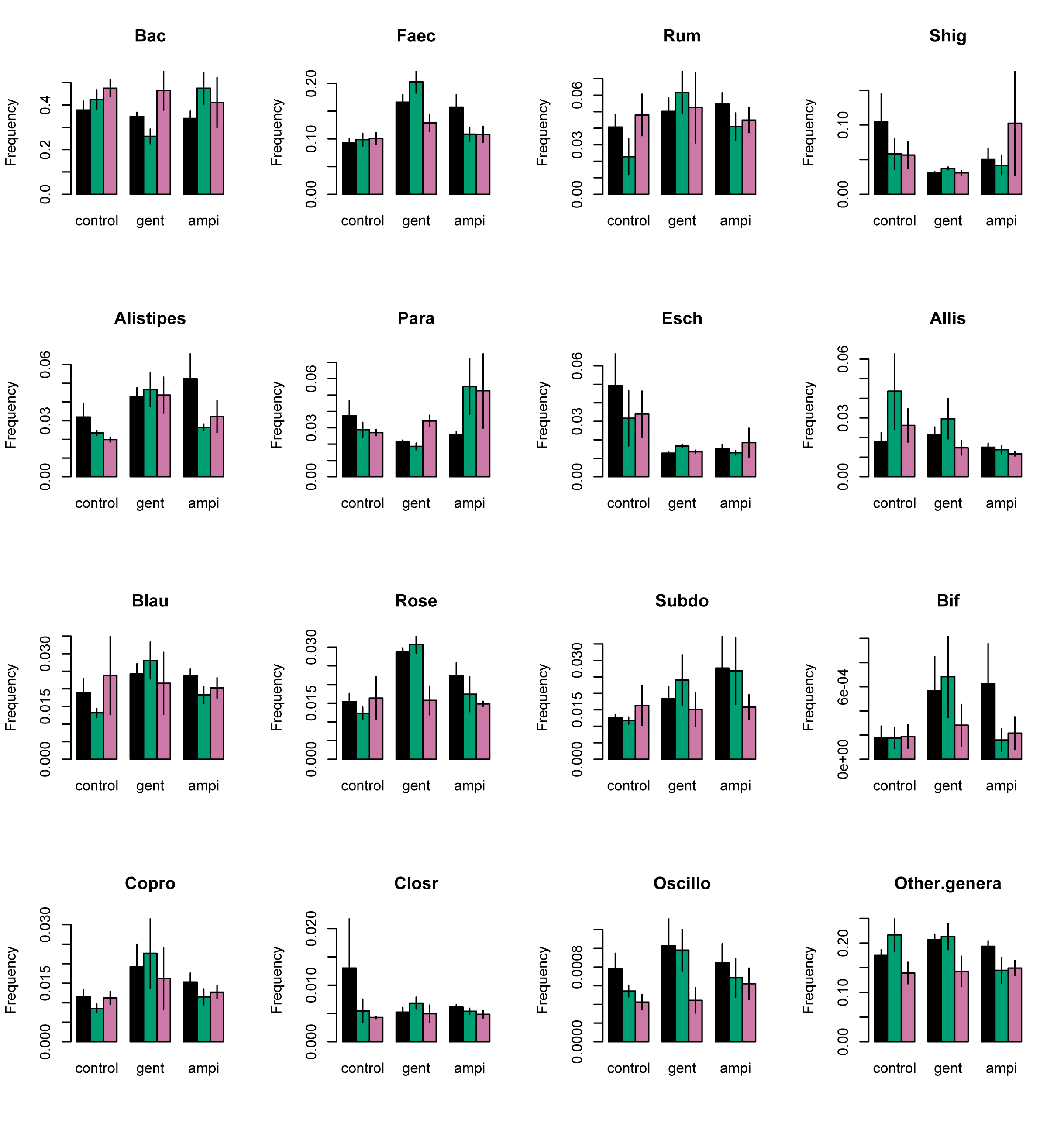


**Figure S1.** Mean frequencies of each taxon determined by Illumina 16S V4 sequencing in each treatment after 10 h. Standard error bars are shown. Black = control; green = inulin;
red = pectin. *Bacteroides* (Bac), *Faecalibacterium* (Faec), *Ruminococcus* (Rum), *Shigella* (Shig), *Parabacteroides* (Para), *Escherichia* (Esch), *Allisonella* (Allis), *Blautia* (Blau), *Roseburia* (Rose), *Subdoligranulum* (Subdo), *Bifidobacterium* (Bif), *Coprococcus* (Copro), *Clostridium* (Closr), *Oscillospira* (Oscillo).


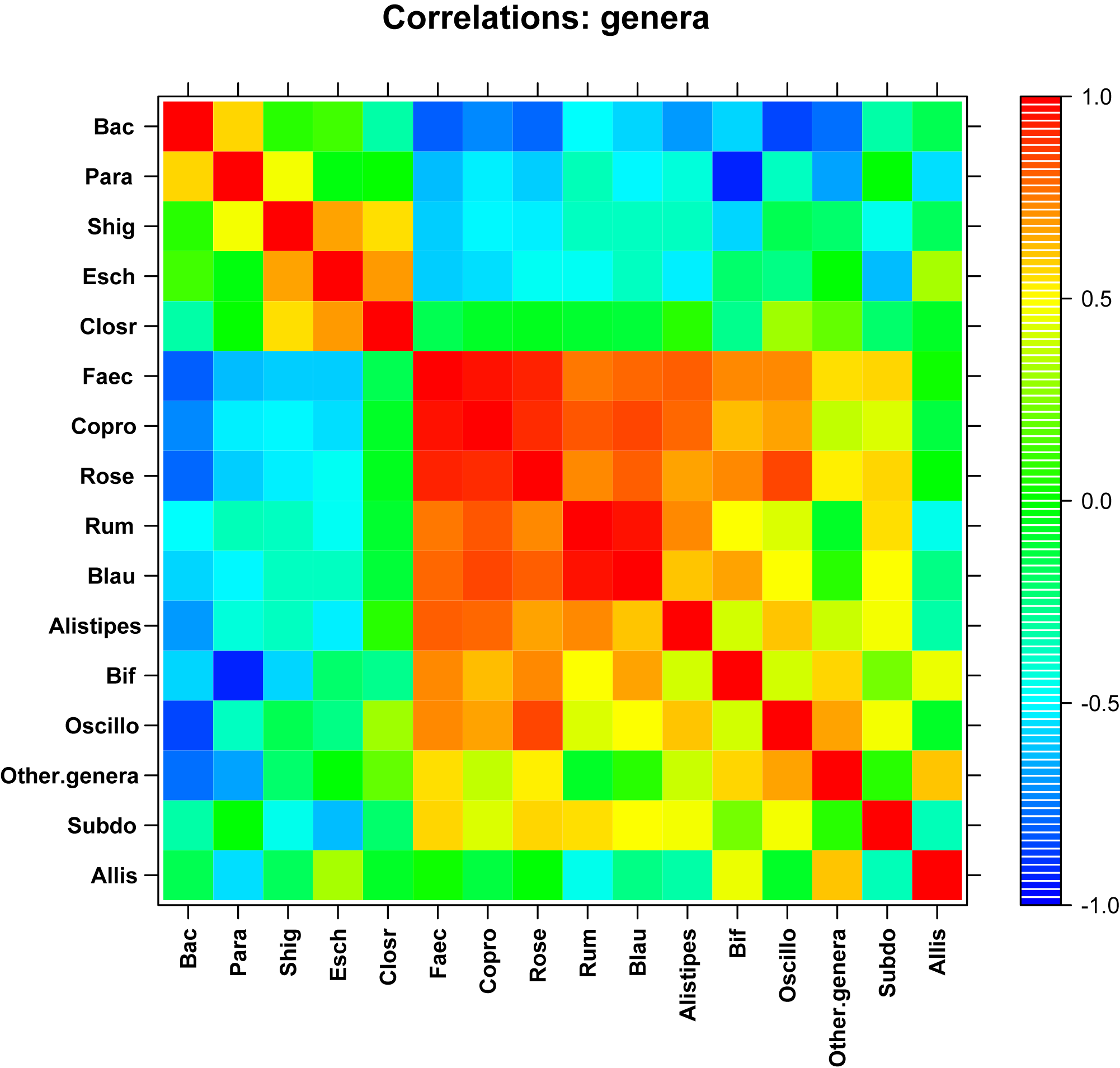


**Figure S2.** Correlations of responses to antibiotic treatment and fermentable fibre supplementation across taxa. Separate linear mixed effect models were fitted for each taxon in turn, and the fixed effects extracted using the ‘effect’ function from the “effects” package in R. A correlation matrix was calculated from a table of the fixed effects across taxa and sorted to place taxa with correlated responses adjacent to one another. Dark red indicates strongly positively correlated responses, dark blue indicates strongly negatively correlated responses. See Figure S1 legend for genera abbreviations.

| 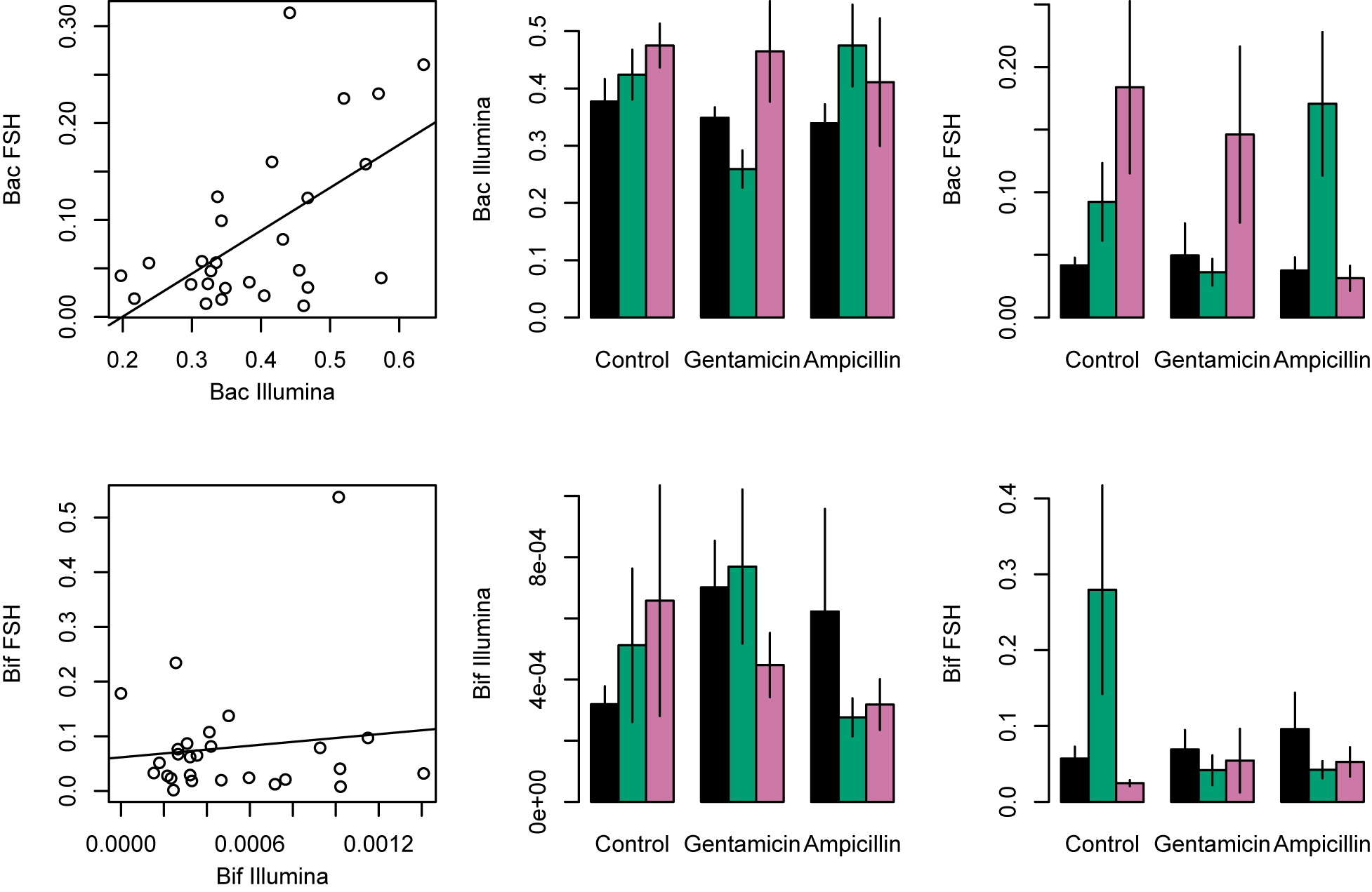 |
| --- |

**Figure S3.** Comparison of frequencies of *Bacteroides* (top row) and *Bifidobacterium* (bottom row) based on FISH counts (relative to total bacteria counts) and Illumina
16S V4 sequencing. There is a significant correlation between the two methods in *Bacteroides* (Bac) (**a**) but not in *Bifidobacterium* (Bif) (**d**). Responses are congruent between the two methods for *Bacteroides* (**b**), (**c**). In a linear mixed effect model with both measures concatenated and microcosm and volunteer as random factors, there was no significant interaction between measurement method with pectin or inulin, antibiotic or their interaction (all *p* > 0.5). In contrast, responses were incongruent for *Bifidobacterium* (**e**), (**f**). There was a significant 3-way interaction between measurement method, fermentable fibre and antibiotics (F_4,33.9_ = 3.33, *p* = 0.021).


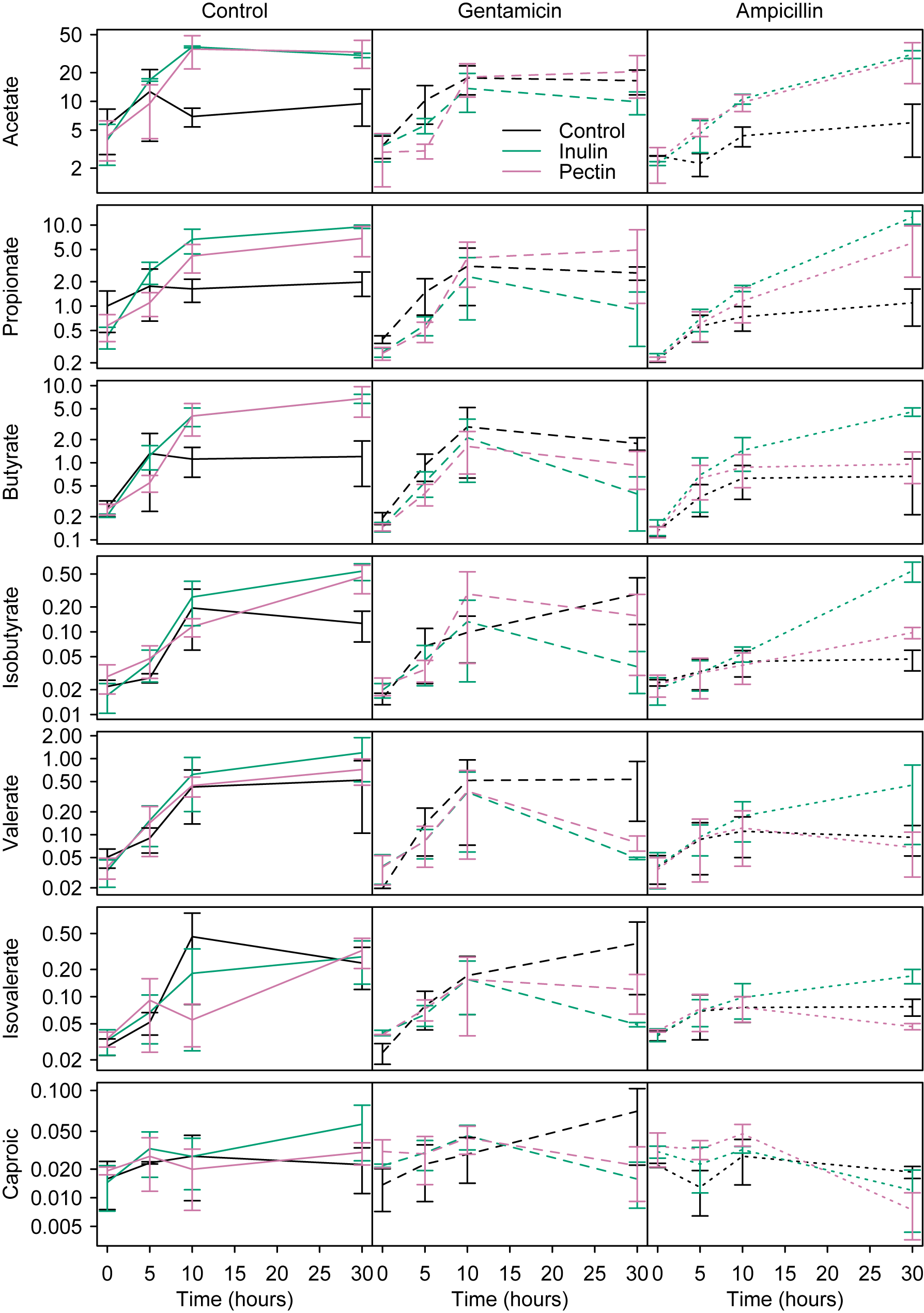


**Figure S4**. Short chain fatty acid (SCFA) log (concentrations) over time. Black = control; green = inulin; pink = pectin. Solid = control antibiotics; dashed = gentamicin;
dotted = ampicillin.

| 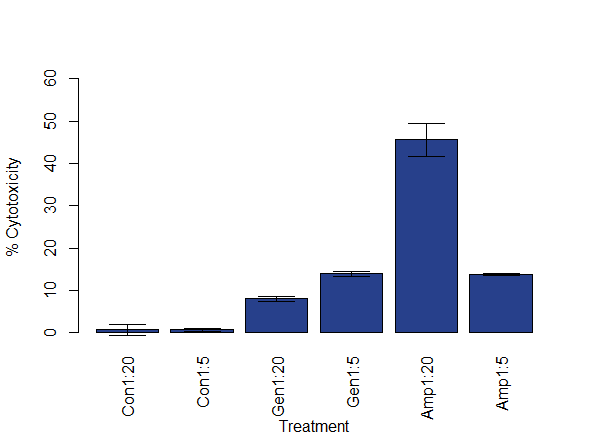 | 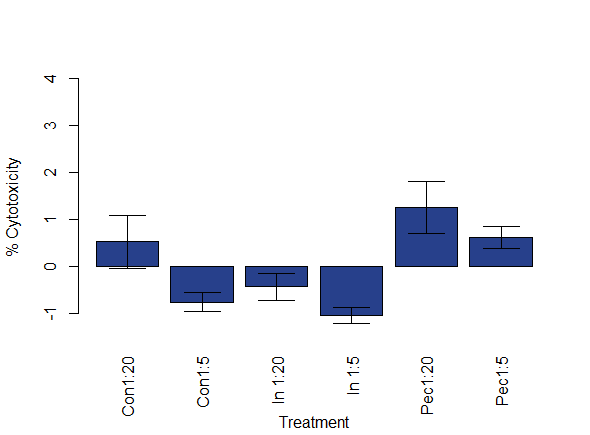 |
| --- | --- |
| **A** | **B** |
| 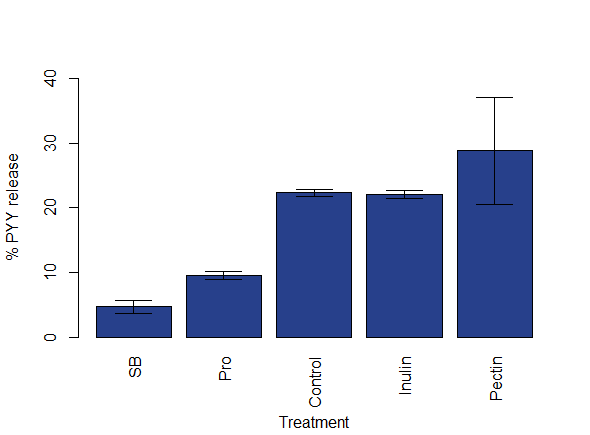 | |
| **C** | |

**Figure S5.** Effects of faecal fluids on colon cell viability and peptide YY (PYY) release. Lactate dehydrogenase (LDH) assay measured cytotoxicity of faecal fluids on colonic
L-cells, with fermentable fibre and antibiotics (**A**) Control, gentamicin and ampicillin at dilutions of 1:20 and 1:5. (**B**) Control, inulin and pectin at dilutions of 1:20 and 1:5. (**C**) PYY secretion from colonic L-cells. Secretion buffer (SB) represents basal release, and propionate (Pro) provides a positive control. Values are means and error bars represent standard error of the mean. Faecal fluid (*t* = 4.7, *p* = 0.0003), faecal fluid supplemented with pectin (*t* = 6.4, *p* ≤ 0.0001) and faecal fluid supplemented with inulin (*t* = 4.6, *p* = 0.0003) caused a significant increase in PYY release compared to the secretion buffer control. However, there was no significant difference between cultures supplemented with pectin or inulin and unsupplemented cultures.

**Table S1**. Effects of treatments on taxon frequencies. Model 0 = no effect; model 1 = both factor levels have same effect; model 2 = factor levels have different effect.

| **Taxon** | **Best.Model** | **Chi.m0m1** | **p1** | **Chi.m1m2** | **p2** |
| --- | --- | --- | --- | --- | --- |
| 1. Dietary supplements in absence of antibiotics |  |  |  |  |  |
| Bacteroides | 2 | 3.736 | 0.053 | 2.304 | 0.129 |
| Faecalibacterium | 0 | 0.858 | 0.354 | 0.078 | 0.780 |
| Ruminococcus | 0 | 0.168 | 0.682 | 3.523 | 0.061 |
| Shigella | 1 | 2.500 | 0.114 | 0.003 | 0.957 |
| Alistipes | 1 | 4.382 | 0.036 | 0.511 | 0.475 |
| Parabacteroides | 1 | 3.398 | 0.065 | 0.120 | 0.729 |
| Escherichia | 0 | 1.604 | 0.205 | 0.027 | 0.870 |
| Allisonella | 0 | 1.813 | 0.178 | 2.023 | 0.155 |
| Blautia | 0 | 0.003 | 0.956 | 1.642 | 0.200 |
| Roseburia | 0 | 0.081 | 0.775 | 0.884 | 0.347 |
| Subdoligranulum | 0 | 0.124 | 0.725 | 1.211 | 0.271 |
| Bifidobacterium | 0 | 1.987 | 0.159 | 0.557 | 0.455 |
| Coprococcus | 0 | 0.849 | 0.357 | 1.995 | 0.158 |
| Clostridium | 1 | 2.167 | 0.141 | 0.037 | 0.847 |
| Oscillospira | 1 | 4.661 | 0.031 | 0.772 | 0.380 |
| Other.genera | 2 | 0.008 | 0.929 | 5.578 | 0.018 |
| 1. Antibiotics in the absence of dietary supplements | |  |  |  |  |
| Bacteroides | 0 | 1.065 | 0.302 | 0.072 | 0.789 |
| Faecalibacterium | 1 | 14.171 | 0 | 0.731 | 0.393 |
| Ruminococcus | 1 | 4.62 | 0.032 | 0.792 | 0.374 |
| Shigella | 1 | 5.381 | 0.02 | 0.571 | 0.45 |
| Alistipes | 2 | 4.886 | 0.027 | 2.446 | 0.118 |
| Parabacteroides | 1 | 4.862 | 0.027 | 0.47 | 0.493 |
| Escherichia | 1 | 7.71 | 0.005 | 0.047 | 0.829 |
| Allisonella | 2 | 0.001 | 0.972 | 5.095 | 0.024 |
| Blautia | 1 | 2.717 | 0.099 | 0.021 | 0.884 |
| Roseburia | 2 | 7.259 | 0.007 | 5.07 | 0.024 |
| Subdoligranulum | 2 | 2.549 | 0.11 | 2.348 | 0.125 |
| Bifidobacterium | 1 | 2.603 | 0.107 | 0.131 | 0.717 |
| Coprococcus | 1 | 2.464 | 0.116 | 1.199 | 0.273 |
| Clostridium | 0 | 1.965 | 0.161 | 0.026 | 0.873 |
| Oscillospira | 0 | 0.706 | 0.401 | 0.765 | 0.382 |
| Other.genera | 1 | 4.41 | 0.036 | 1.681 | 0.195 |

**Table S2.** Effects of antibiotics treatments on taxon counts (calculated by multiplying frequencies estimated by 16S rDNA sequencing with total bacteria FSH counts). Model 0 = no effect; model 1 = both factor levels have same effect; model 2 = factor levels have different effect.

|  | **Best.model** | **Chi.m0m1** | **p1** | **Chi.m1m2** | **p2** |
| --- | --- | --- | --- | --- | --- |
| Bacteroides | 1 | 4.36 | 0.037 | 0.04 | 0.842 |
| Faecalibacterium | 1 | 2.21 | 0.137 | 0.001 | 0.981 |
| Ruminococcus | 0 | 0.058 | 0.81 | 0.113 | 0.737 |
| Shigella | 1 | 7.614 | 0.006 | 0.34 | 0.56 |
| Alistipes | 0 | 0.329 | 0.566 | 1.328 | 0.249 |
| Parabacteroides | 1 | 9.352 | 0.002 | 0.375 | 0.54 |
| Escherichia | 1 | 6.95 | 0.008 | 0.015 | 0.903 |
| Allisonella | 0 | 1.302 | 0.254 | 0.88 | 0.348 |
| Blautia | 0 | 0.181 | 0.671 | 0.007 | 0.935 |
| Roseburia | 0 | 0.725 | 0.394 | 0.969 | 0.325 |
| Subdoligranulum | 0 | 0.729 | 0.393 | 1.876 | 0.171 |
| Bifidobacterium | 0 | 0.655 | 0.418 | 0.476 | 0.49 |
| Coprococcus | 0 | 0.128 | 0.721 | 0.354 | 0.552 |
| Clostridium | 1 | 2.845 | 0.092 | 0.014 | 0.905 |
| Oscillospira | 0 | 0.145 | 0.703 | 0.219 | 0.64 |
| Other.genera | 0 | 0.554 | 0.457 | 0.155 | 0.694 |

**Table S3.** Comparison of models with dietary supplementation, antibiotic and their interaction fitted to the frequencies of each genus in turn. Model 1 = diet × antibiotic interaction; Model 2 = diet + antibiotic; Model 3 = just dietary supplementation;
Model 4 = just antibiotics; Model 5 = just intercept, no treatments significant.

|  | **Chi Diet** | **P Diet** | **Chi Antibiotic** | **P Antibiotic** | **Chi Interaction** | **P Interaction** | **Best Model** |
| --- | --- | --- | --- | --- | --- | --- | --- |
| Bacteroides | 4.085 | 0.13 | 2.235 | 0.327 | 7.547 | 0.11 | 5 |
| Faecalibacterium | 4.99 | 0.083 | 20.121 | 0 | 20.605 | 0 | 1 |
| Ruminococcus | 1.014 | 0.602 | 4.945 | 0.084 | 5.521 | 0.238 | 4 |
| Shigella | 0.794 | 0.672 | 3.494 | 0.174 | 4.826 | 0.306 | 5 |
| Alistipes | 7.033 | 0.03 | 15.385 | 0 | 11.341 | 0.023 | 1 |
| Parabacteroides | 1.607 | 0.448 | 6.035 | 0.049 | 7.807 | 0.099 | 4 |
| Escherichia | 0.762 | 0.683 | 13.803 | 0.001 | 2.91 | 0.573 | 4 |
| Allisonella | 5.571 | 0.062 | 7.951 | 0.019 | 5.866 | 0.209 | 2 |
| Blautia | 0.451 | 0.798 | 2.268 | 0.322 | 3.838 | 0.428 | 5 |
| Roseburia | 5.522 | 0.063 | 12.09 | 0.002 | 11.841 | 0.019 | 1 |
| Subdoligranulum | 1.938 | 0.379 | 6.174 | 0.046 | 5.246 | 0.263 | 4 |
| Bifidobacterium | 0.213 | 0.899 | 2.085 | 0.353 | 5.093 | 0.278 | 5 |
| Coprococcus | 0.499 | 0.779 | 8.793 | 0.012 | 2.482 | 0.648 | 4 |
| Clostridium | 2.335 | 0.311 | 1.085 | 0.581 | 4.412 | 0.353 | 5 |
| Oscillospira | 9.43 | 0.009 | 3.88 | 0.144 | 3.979 | 0.409 | 3 |
| Other genera | 8.822 | 0.012 | 2.095 | 0.351 | 7.545 | 0.11 | 3 |

© 2015 by the authors; licensee MDPI, Basel, Switzerland. This article is an open access article distributed under the terms and conditions of the Creative Commons Attribution license (http://creativecommons.org/licenses/by/4.0/).
